# Intravascular Leukocyte Labeling Refines the Distribution of Myeloid Cells in the Lung in Models of Allergen-induced Airway Inflammation

**DOI:** 10.4049/immunohorizons.2300059

**Published:** 2023-12-15

**Authors:** Yu-Hua Chow, Ryan C. Murphy, Dowon An, Ying Lai, William A. Altemeier, Anne M. Manicone, Teal S. Hallstrand

**Affiliations:** Division of Pulmonary, Critical Care, and Sleep Medicine and Center for Lung Biology, Department of Medicine, University of Washington, Seattle, WA 98109

## Abstract

Innate immune cell populations are critical in asthma with different functional characteristics based on tissue location, which has amplified the importance of characterizing the precise number and location of innate immune populations in murine models of asthma. In this study, we performed premortem intravascular (IV) labeling of leukocytes in mice in two models of asthma to differentiate innate immune cell populations within the IV compartment versus those residing in the lung tissue or airway lumen. We performed spectral flow cytometry analysis of the blood, suspensions of digested lung tissue, and bronchoalveolar lavage fluid. We discovered that IV labeled leukocytes do not contaminate analysis of bronchoalveolar lavage fluid but represent a significant proportion of cells in digested lung tissue. Exclusion of IV leukocytes significantly improved the accuracy of the assessments of myeloid cells in the lung tissue and provided important insights into ongoing trafficking in both eosinophilic and neutrophilic asthma models.

## Introduction

Murine models of asthma have become increasingly sophisticated, progressing from the initial T cell–dependent models to inhaled complete allergen models that do not require an adjuvant and are not T cell–dependent, although they involve specific T cell subsets (1). These models better recapitulate many features of inflammation implicated in asthma and recently have been extended to both type 2 and non–type 2 models of airway inflammation (2–4). Although resident and recruited T cells play a critical role in each of these models, myeloid and innate immune cells also play major roles in disease pathogenesis within these model systems and within human asthma (5–9).

Premortem intravascular (IV) labeling of specific immune cell populations prior to harvesting murine lung tissue is well-established in studies of T cell biology and has been used effectively to distinguish between lung resident T cell populations from circulating and recruited populations (10–13). Although IV labeling has been used to study trafficking of myeloid cell populations to the lungs in lung injury models (14, 15), IV leukocyte labeling has not been used to define the distinct tissue compartment location of innate immune populations in modern murine models of asthma. The distinction between resident and circulating innate immune cell populations is becoming increasingly important in understanding key inflammatory mechanisms present in asthma, because recent studies have indicated that there may be significant functional differences in these cell populations (16).

In this study, we performed two murine models of asthma, one using a traditional airway proteolytic allergen sensitization and challenge and another utilizing peripheral administration of CFA to promote an enhanced type 1–mediated response and used spectral flow cytometry with premortem IV labeling of CD45^+^ cells to determine the composition of individual leukocyte populations within the blood, IV compartment of the lung, the lung tissue, and the airway lumen. We also developed a broad cell surface marker panel to characterize the composition of immune cells, which included the positive identification of both eosinophils and neutrophils. We examined the effects of ex vivo exclusion of circulating cells on the interpretation of leukocyte populations within the lung tissue and airway lumen, confirming prior work demonstrating that postmortem perfusion of the pulmonary vasculature does not remove IV leukocytes from the lung tissue and that lymphocyte populations represent a majority of the leukocytes that persist within the pulmonary vasculature (11, 15). However, we also demonstrated that removal of these IV labeled leukocytes dramatically alters the measured composition of leukocyte populations in the lung tissue, which better clarifies the tissue compartmental immune response and highlights the role of myeloid populations in models of allergen-induced airway inflammation. Finally, by measuring the composition of cells across multiple tissue compartments, we were able to define trafficking patterns of individual cell populations.

## Materials and Methods

### Mice and models of asthmatic airway inflammation

Wild type C57BL/6 mice were used in all experiments and were obtained from The Jackson Laboratory. Animals were maintained under specific pathogen-free conditions at the University of Washington and had ad libitum access to food and water. Female mice between 6 and 12 wk old were used in experiments to reduce heterogeneity. The University of Washington Institutional Animal Care and Use Committee approved all animal studies. To induce allergic airway disease, the mice were administered house dust mite (HDM) extract (catalog no. RMB84M, 275533; Greer Laboratories) or saline (as a control condition) by oropharyngeal aspiration on days 1 and 14 (100 μg/day) and days 26–28 (50 μg/day) followed by collection on day 29 (Supplemental Fig. 2). To model neutrophilic airway inflammation, mice were sensitized with a s.c. injection of HDM (200 μg) mixed 1:1 by volume with CFA (Sigma-Aldrich, catalog no. F5881) at the base of the tail and subsequently challenged with oropharyngeal HDM extract (100 μg) on day 13 followed by collection on day 14, similar to a previously described protocol (Supplemental Fig. 2) (3, 4).

### Intravascular labeling procedure, sample collection, and processing

The mice were anesthetized with isoflurane and received retro-orbital injection of an anti-CD45 antibody (AF488-CD45.2, BioLegend, catalog no. 109816, 0.5 mg/ml). Three minutes following retro-orbital injection, the mice were euthanized via i.p. injection of Euthasol (Virbac AH, Fort Worth, TX). Sternotomy was performed, and blood was collected from the right ventricle. Tracheostomy was performed, and bronchoalveolar lavage (BAL) was performed as previously described (17). The abdominal aorta was transected, and 5 ml of cold HBSS was injected into the right ventricle to flush the pulmonary vasculature. The left lung was removed and processed into a single cell suspension as previously described (17).

### Spectral flow cytometry of leukocyte populations

We used a 10-fluorochrome panel for cell staining and gating strategy that builds upon a previously described panel (18) but with modifications to include positive selection of eosinophils with SiglecF and with T cell confirmation by CD3e (Supplemental Fig. 1). Briefly, leukocyte populations isolated from the peripheral blood, single cell suspensions of lung tissue, and BAL fluid were stained for cell surface markers as previously described with the following fluorochrome-conjugated Abs: fixed viability dye–eFlour 780 (eBioscience, catalog no. 65-0865-14), CD45.1-PerCP (BioLegend, catalog no. 103130), Ly6G-APC (eBioscience, catalog no. 17-5931-82), CD11b-AF700 (BioLegend, catalog no. 101222), CD11c-PE/Cy7 (BioLegend, catalog no. 117318), MHCII-BUV395 (BD Biosciences, catalog no. 743876), CD24-BV421 (BioLegend, catalog no. 101826), CD3e-PE/Dazzle594 (BioLegend, catalog no. 100348), SiglecF-PE (BD Biosciences, catalog no. 552126), CD64-BV711 (BioLegend, catalog no. 139311), and Ly6C-BV650 (BioLegend, catalog no. 128049). The cells were analyzed using a five-laser Aurora spectral flow cytometer (Cytek Biosciences), and the data were analyzed using FlowJo software (TreeStar). Our gating strategy began with size and granularity exclusion of squamous epithelial cells and cellular debris followed by removal of doublets followed by removal of dead cells (not shown). Among the live cell populations, total CD45^+^ cells were identified first (CD45.1^+^), followed by characterization of leukocytes as either IV (CD45.2^+^) or lung tissue cells (CD45.2^−^). Individual cell populations were defined as follows: neutrophils (Ly6G^+^), T cells (Ly6G^−^CD11b^−^CD11c^-^MHCII^lo^CD24^−^CD3e^+^), B cells (Ly6G^−^CD11b^−^CD11c^−^MHCII^+^CD24^+^), eosinophils (Ly6G^−^CD11b^+^CD11c^int^MHCII^lo^SiglecF^+^), MHCII^lo^ macrophages (Ly6G^−^CD11b^−^CD11c^+^MHCII^lo^CD64^+^), monocytes (Ly6G^−^CD11b^+^CD11c^+^MHCII^lo^CD64^+^), NK cells (Ly6G^−^CD11b^int^CD11c^+^MHCII^lo^CD64^−^), MHCII^high^ macrophages (Ly6G^−^CD11b^+^CD11c^+^MHCII^high^CD64^+^CD24^int^), and dendritic cells (Ly6G^−^CD11b^+^MHCII^+^CD64^int^CD24^+^).

### Data analysis

For comparisons of individual cell population percentages from mice sensitized and challenged with either HDM extract alone or HDM extract/CFA versus saline controls, *p* values were calculated using an unpaired two-tailed Student *t* test. For comparisons of individual cell population percentages in the blood versus the IV compartment of the lung tissue, *p* values were calculated using a two-way ANOVA with correction for multiple comparisons using the two-stage step-up method of Benjamini, Krieger, and Yekutieli. For comparisons of individual cell population percentages or total cell counts with and without exclusion of IV CD45.2^+^ cells, *p* values were calculated using a paired two-tailed Student *t* test. A *p* value less than 0.05 was considered significant.

## Results

### Intravascular leukocyte labeling has a significant impact on the assessment of lung leukocytes

**FIGURE 1.**
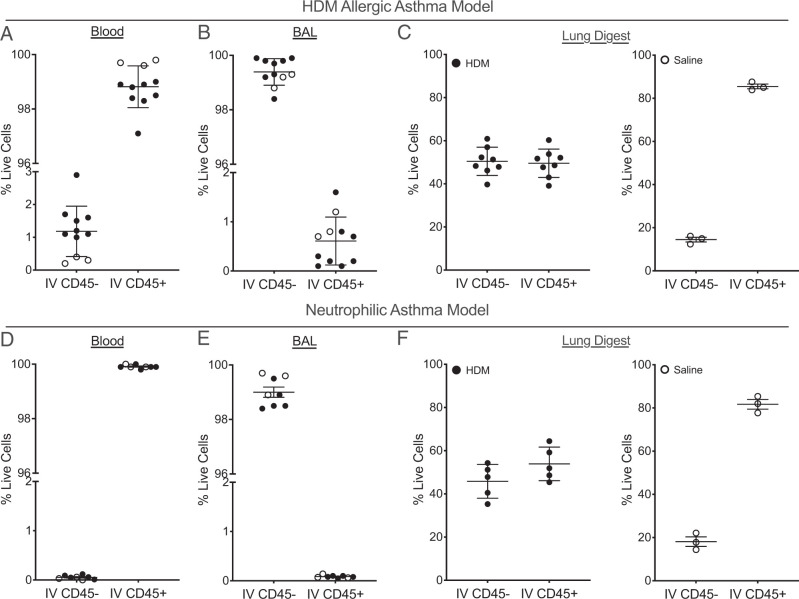
Proportion of cells labeled with the IV label in the different compartments in two different models of asthma. (**A** and **D**) In both models, the IV CD45.2 label administered by retro-orbital injection 3 min before euthanasia labeled most leukocytes in the blood, with or without sensitization and challenge. (**B** and **E**) At this early time point, the IV CD45.2-labeled cells do not appear in the BAL fluid. (**C** and **F**) Without sensitization and challenge, the majority of leukocytes that appear in the lung contain the IV CD45.2 label, whereas a smaller number of resident cells do not have the IV CD45.2 label. Mean values are shown with error bars representing the SD.

Administration of the IV CD45.2 Ab by retro-orbital injection 3 min before sternotomy and sample collection was highly effective at labeling circulating leukocytes (Fig. 1A, 1D). We were only able to detect background levels of IV CD45.2^+^ cells in BAL fluid in both sensitized and unsensitized mice, indicating that circulating cell populations labeled just prior to euthanasia and BAL collection do not enter the airway lumen and have no impact on the evaluation of immune cell differentials in BAL fluid (Fig. 1B, 1E). In contrast, a significant portion of leukocytes harvested from digested lung tissue were IV CD45.2^+^, despite flushing the pulmonary vasculature prior to generating single cell suspensions (Fig. 1C, 1F). Approximately half of the lung leukocytes were IV CD45.2^+^ in sensitized mice from both models, whereas most lung leukocytes were IV CD45.2^+^ in unsensitized mice from both models. These findings indicate that leukocytes residing in the vascular compartment are still present in single cell suspensions of lung tissue despite flushing of the pulmonary vasculature (10). These IV CD45.2^+^ cells represent marginated cells adherent to the endothelial wall or cells that are sequestered or have already trafficked to specific areas of inflamed lung tissue or entered the lung tissue via regions of endothelial damage during flushing of the pulmonary vasculature (14, 15, 19, 20). However, our results indicate that circulating and vascular adherent leukocyte populations are mixed with both resident and recruited cell populations in lung tissue, which may affect the interpretation of leukocyte populations in the lung tissue and limit our ability to understand cell-specific trafficking patterns.

### Intravascular leukocyte labeling improves the accuracy of characterizing myeloid cells in the lung

**FIGURE 2.**
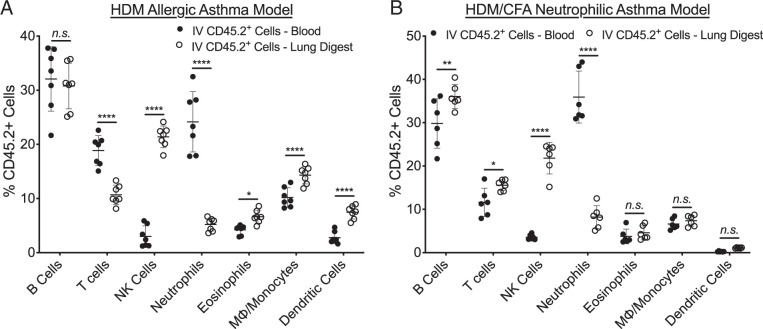
Composition of IV labeled leukocytes in the blood and digested lung tissue in two different models of asthma. (**A**) In mice receiving airway sensitization and challenge with HDM extract in our HDM allergic asthma model, IV labeled leukocytes (CD45.2^+^) identified in the blood had similar composition to IV labeled leukocytes in digested lung tissue, consisting primarily of lymphocyte populations and neutrophils. (**B**) We also saw a similar pattern in mice receiving sensitization and challenge with HDM extract and CFA in our HDM/CFA neutrophilic asthma model. Mean values are shown with error bars representing the SD. For comparisons of individual cell population percentages in the blood versus IV compartment of the lung tissue, *p* values were calculated using a two-way ANOVA with correction for multiple comparisons using the two-stage step-up method of Benjamini, Krieger, and Yekutieli. *, *p* < 0.05, **, *p* < 0.01; ***, *p* < 0.001; ****, *p* < 0.0001.

**FIGURE 3.**
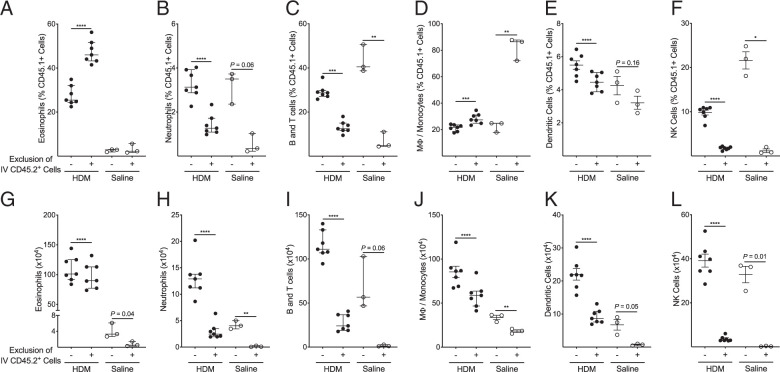
Exclusion of IV CD45.2-labeled cells changed the proportion and absolute number of leukocytes in the lung tissue compartment in our HDM allergic asthma model. (**A–F**) Among the total CD45.1^+^, exclusion of CD45.2^+^ cells resulted in a significant increase in the proportion of eosinophils (A) and macrophages/monocytes (D) and a decrease in the proportion of neutrophils (B), B and T cells (C), dendritic cells (E), and NK cells (F). (**G–L**) Exclusion of CD45.2^+^ cells resulted in a modest but significant decrease in total eosinophils (G) and otherwise resulted in a significant decrease in the total number of neutrophils (H), B and T cells (I), macrophages/monocytes (J), dendritic cells (K), and NK cells (L). Median values are shown with error bars representing the interquartile range. For comparisons of individual cell population percentages or total cell counts with and without exclusion of IV CD45.2^+^ cells, *p* values were calculated using a paired two-tailed Student *t* test. *, *p* < 0.05; **, *p* < 0.01; ***, *p* < 0.001; ****, *p* < 0.0001.

**FIGURE 4.**
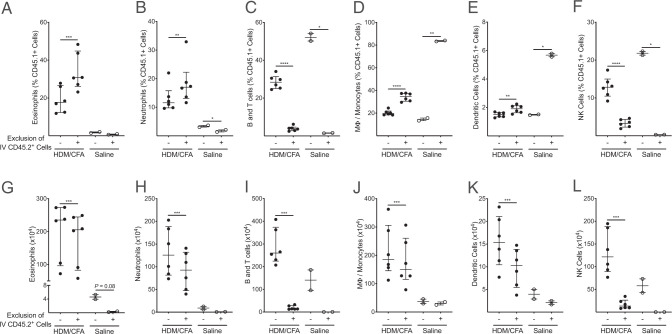
Exclusion of IV CD45.2-labeled cells changed the proportion and absolute number of leukocytes in the lung tissue compartment in our HDM/CFA neutrophilic asthma model. (**A–F**) Among the total CD45.1^+^, exclusion of CD45.2^+^ cells resulted in a significant increase in the proportion of eosinophils (A), neutrophils (B), macrophages/monocytes (D), and dendritic cells (E) and a decrease in the proportion of B cells and T cells (C) and NK cells (F). (**G–L**) Exclusion of CD45.2^+^ cells resulted in a significant decrease in the total numbers of cells in every leukocyte population, but there was a larger numerical decrease in the number of B cells and T cells (I) and NK cells (L) relative to eosinophils (G), neutrophils (H), macrophages/monocytes (J), and dendritic cells (K). Median values are shown with error bars representing the interquartile range. For comparisons of individual cell population percentages or total cell counts with and without exclusion of IV CD45.2^+^ cells, *p* values were calculated using a paired two-tailed Student *t* test. *, *p* < 0.05; **, *p* < 0.01; ***, *p* < 0.001; ****, *p* < 0.0001.

The composition of the IV labeled leukocytes identified in the lung tissue is similar to the composition of the circulating leukocyte population (Fig. 2, Supplemental Fig. 3). The peripheral blood is primarily composed of lymphocyte populations (B cells, T cells, and NK cells) and neutrophils. The lung IV CD45.2^+^ cells had a lower percentage of neutrophils and higher percentage of NK cells in comparison with the peripheral blood in both models. Removal of IV leukocytes provides an opportunity to focus solely on lung tissue leukocytes, which significantly affects the composition and total number of the measured immune cell populations (Fig. 3, 4). In the HDM allergic asthma model, eosinophils (Fig. 3A) and macrophages/monocytes (Fig. 3D) represented a significantly larger proportion of the total leukocyte population among the lung tissue resident cells, whereas neutrophils (Fig. 3B), B and T cells (Fig. 3C), dendritic cells (Fig. 3E), and NK cells (Fig. 3F) represented a significantly smaller proportion. Excluding IV labeled leukocytes from our analysis of the leukocytes in the lung tissue resulted in a significant reduction in the total number of cells identified in all measured cell populations (Fig. 3G–L). However, there were numerically smaller decreases in the total number of eosinophils (Fig. 3G) and macrophages/monocytes (Fig. 3J), whereas there were dramatic decreases in the numbers of neutrophils (Fig. 3H), B and T cells (Fig. 3I), dendritic cells (Fig. 3K), and NK cells (Fig. 3L). In the HDM/CFA neutrophilic asthma model, there is a similar pattern with an increased proportion of eosinophils (Fig. 4A) and macrophages/monocytes (Fig. 4D) and a decreased proportion of B and T cells (Fig. 4C) and NK cells (Fig. 4F). In contrast to the HDM allergic asthma model, there is an increase in the proportion of neutrophils (Fig. 4B) and dendritic cells (Fig. 4E) among the lung tissue cells. Overall, lymphocyte populations (B, T, and NK cells) represented the majority of the IV labeled leukocytes in both models, and importantly, exclusion of these leukocytes allowed for more accurate representation of innate immune cell populations among the lung tissue leukocytes in both the HDM allergic asthma and HDM/CFA neutrophilic asthma models.

### Intravascular labeling can be used to assess the ongoing trafficking of myeloid cells to the lung

**FIGURE 5.**
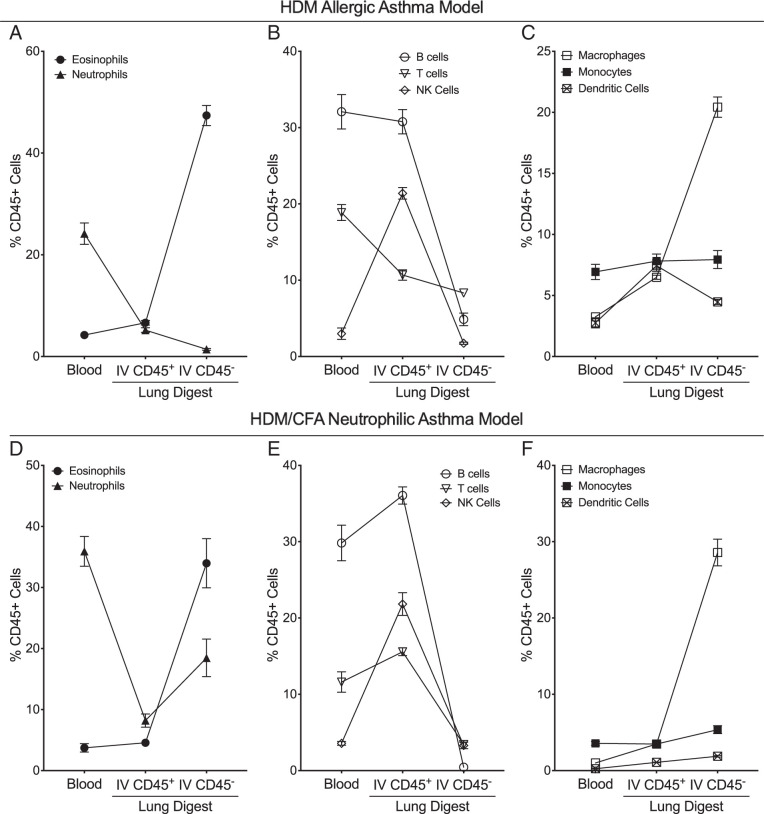
IV leukocyte labeling allows for identification of cell trafficking patterns into the lung. (**A–C**) In our HDM allergic asthma model, there was an increase in the percentage of eosinophils (A) and macrophages (C) in the lung tissue compartment (IV CD45.2^−^ cells) relative to the IV space in the lungs (IV CD45.2^+^ cells) and the blood. There was a consistent decrease in the percentage of neutrophils (A), B cells (B), and T cells (B) from the blood to the IV compartment of the lung to the lung tissue. NK cells (B) were overrepresented in the IV compartment of the lung relative to the blood or lung tissue. Monocytes and dendritic cells (C) were represented in similar proportions in the IV compartment of the lung and the lung tissue. (**D–F**) In our HDM/CFA neutrophilic asthma model, there was a similar increase in the percentage of eosinophils (D) and macrophages (F) and a decrease in the percentage of lymphocyte populations (E) in the lung tissue compartment (IV CD45.2^−^ cells) relative to the IV space in the lungs (IV CD45.2^+^ cells). In contrast to the HDM allergic asthma model, we identified an increase in the percentage of neutrophils (E) in the lung tissue compartment (IV CD45.2^−^ cells) relative to the IV space in the lungs (IV CD45.2^+^ cells). The percentage of individual cell populations represents the percentage of total cells within each compartment (i.e., IV CD45^+^ eosinophils represent the percentage of eosinophils among the total IV CD45.2^+^ positive population, and IV CD45^−^ eosinophils represent the percentage of eosinophils among the total IV CD45.2^−^ negative population). Mean values are shown with error bars representing the SEM.

An additional advantage of labeling IV leukocytes is the ability to identify trafficking patterns of specific cell populations to the lung versus cell populations that are primarily present in the pulmonary vasculature and may contaminate the profiling of lung tissue leukocytes in both sensitized and saline control mice (15). Among sensitized mice from both the HDM allergic asthma model and the HDM/CFA neutrophilic asthma models, eosinophils were low in both the peripheral blood and the IV compartment of the lung but were significantly elevated in the lung tissue compartment (Fig. 5A, 5D). In contrast, lymphocytes (Fig. 5B, 5E) were elevated in both the peripheral blood and the IV compartment of the lung but comprised a lower proportion of lung tissue leukocytes. Thus, eosinophils demonstrated a pattern consistent with trafficking to the lung tissue whereas lymphocyte populations are primarily present in the vasculature in both models of airway inflammation. However, we did observe difference patterns of neutrophil trafficking between our two model systems. In the HDM allergic asthma model, neutrophils consistently declined in the proportion of leukocytes from the blood to the lung tissue (Fig. 5A), suggesting that neutrophils are primarily present in the vasculature in this model. In the HDM/CFA neutrophilic asthma model, the proportion of neutrophils in the IV compartment of the lung was relatively reduced compared with the peripheral blood but compromised a larger percentage of leukocytes within the lung tissue (Fig. 5D). These findings highlight key differences in compartmental immune responses between these two model systems, notably that neutrophils demonstrate a trafficking pattern to the lung tissue in the HDM/CFA model. However, it is important to note that the HDM/CFA model system also induces considerable eosinophil trafficking to the lung tissue and is likely best characterized as a model of mixed eosinophilic and neutrophilic airway inflammation. Mononuclear cell populations demonstrated similar patterns of leukocyte composition between compartments in both model systems, including a higher percentage of macrophages within the lung tissue and overall similar percentages of monocytes and dendritic cells in the IV compartment of the lung and lung tissue (Fig. 5C, 5F).

## Discussion

IV leukocyte labeling is an effective method to characterize leukocyte populations present within the lung tissue compartment with greater accuracy. Leukocytes that reside within the pulmonary vasculature represent a significant proportion of the total leukocytes in lung digest tissue and persist despite flushing the pulmonary vasculature. In two distinct murine models of asthma, we found that exclusion of IV labeled leukocytes markedly altered the proportion of myeloid cells among lung tissue leukocytes. Finally, IV leukocyte labeling and separate characterization of leukocyte proportions in the blood, IV compartment of the lung, and lung tissue can reveal patterns of leukocyte trafficking among innate immune cell populations, which can serve as a useful tool to identify specific immune cell subpopulations within distinct tissue compartments.

## Supplementary Material

Supplemental Figures 1 (PDF)
