# Supplementary material for: Intravascular Leukocyte Labeling Refines the Distribution of Myeloid Cells in the Lung in Models of Allergen-induced Airway Inflammation
Source: Immunohorizons. 2023 Dec 15;7(12):853–60. doi: 10.4049/immunohorizons.2300059 (PMC10759158; doi:10.4049/immunohorizons.2300059)
Supplement: Supplemental Figures 1 (PDF) [file IH_2300059_Supplemental_1.pdf]

SUPPLEMENTAL FIGURES & LEGENDS

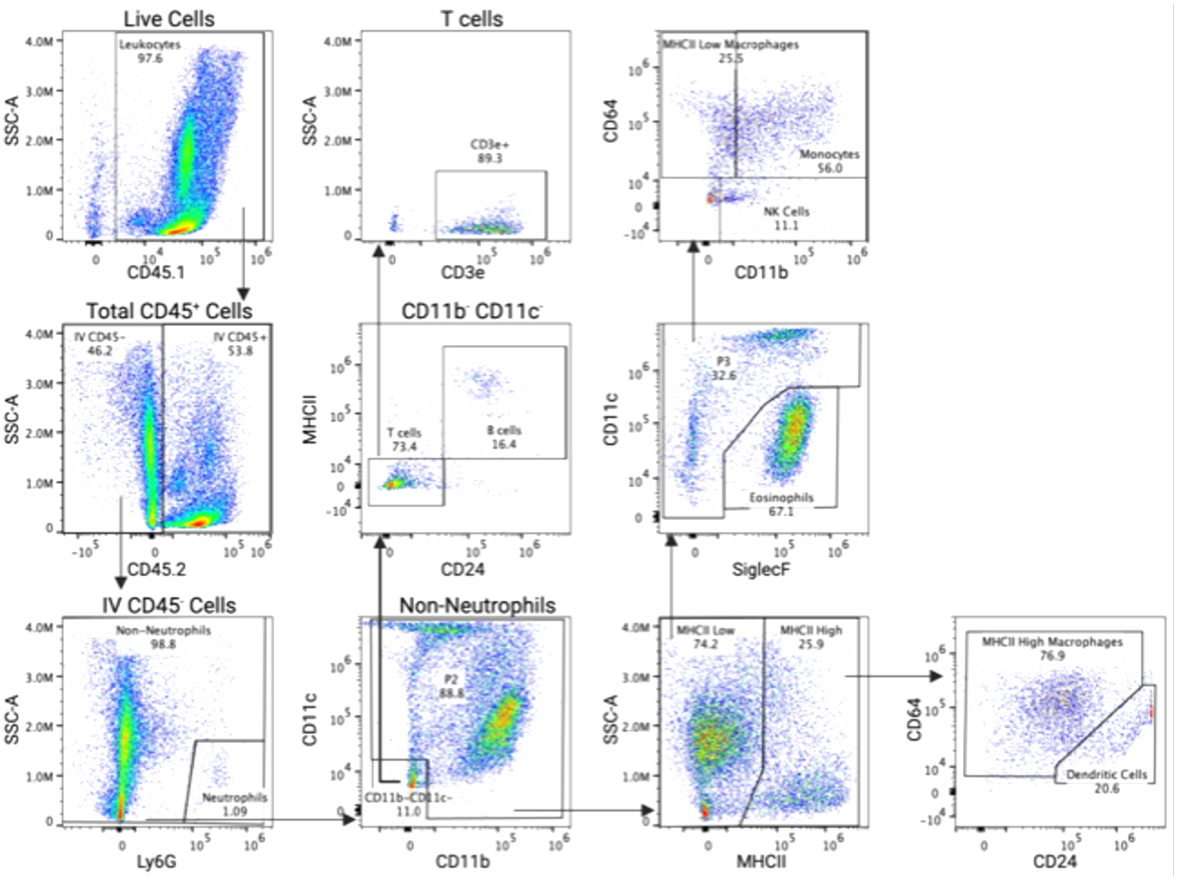

Supplemental Figure 1. Spectral flow cytometry gating strategy. Our gating strategy began with size and granularity exclusion of squamous epithelial cells and cellular debris followed by removal of doublets followed by removal of dead cells (not shown). Amongst the live cell populations, total CD45^{+} cells were identified first (CD45.1^{+}), followed by characterization of leukocytes as either intravascular (CD45.2^{+}) or lung tissue resident cells (CD45.2^{-}). Individual cell populations were defined as follows: neutrophils (Ly6G^{+}SSC^{lo}), T cells (Ly6G^{-}CD11b^{-}CD11c^{-}MHCII^{lo}CD24^{-}CD3e^{+}), B cells (Ly6G^{-}CD11b^{-}CD11c^{-}MHCII^{+}CD24^{+}), eosinophils (Ly6G^{-}CD11b^{+}CD11c^{int}MHCII^{lo}SiglecF^{+}), alveolar macrophages which include MHCII^{lo} macrophages (Ly6G^{-}CD11b^{-}CD11c^{+}MHCII^{lo}CD64^{+}) and MHCII^{high} macrophages (Ly6G^{-}CD11b^{+}CD11c^{+}MHCII^{high}CD64^{+}CD24^{int}), monocytes (Ly6G^{-}CD11b^{+}CD11c^{+}MHCII^{lo}CD64^{+}), NK cells (Ly6G^{-}CD11b^{int}CD11c^{+}MHCII^{lo}CD64^{-}), and dendritic cells (Ly6G^{-}CD11b^{+}MHCII^{+}CD64^{int}CD24^{+}).

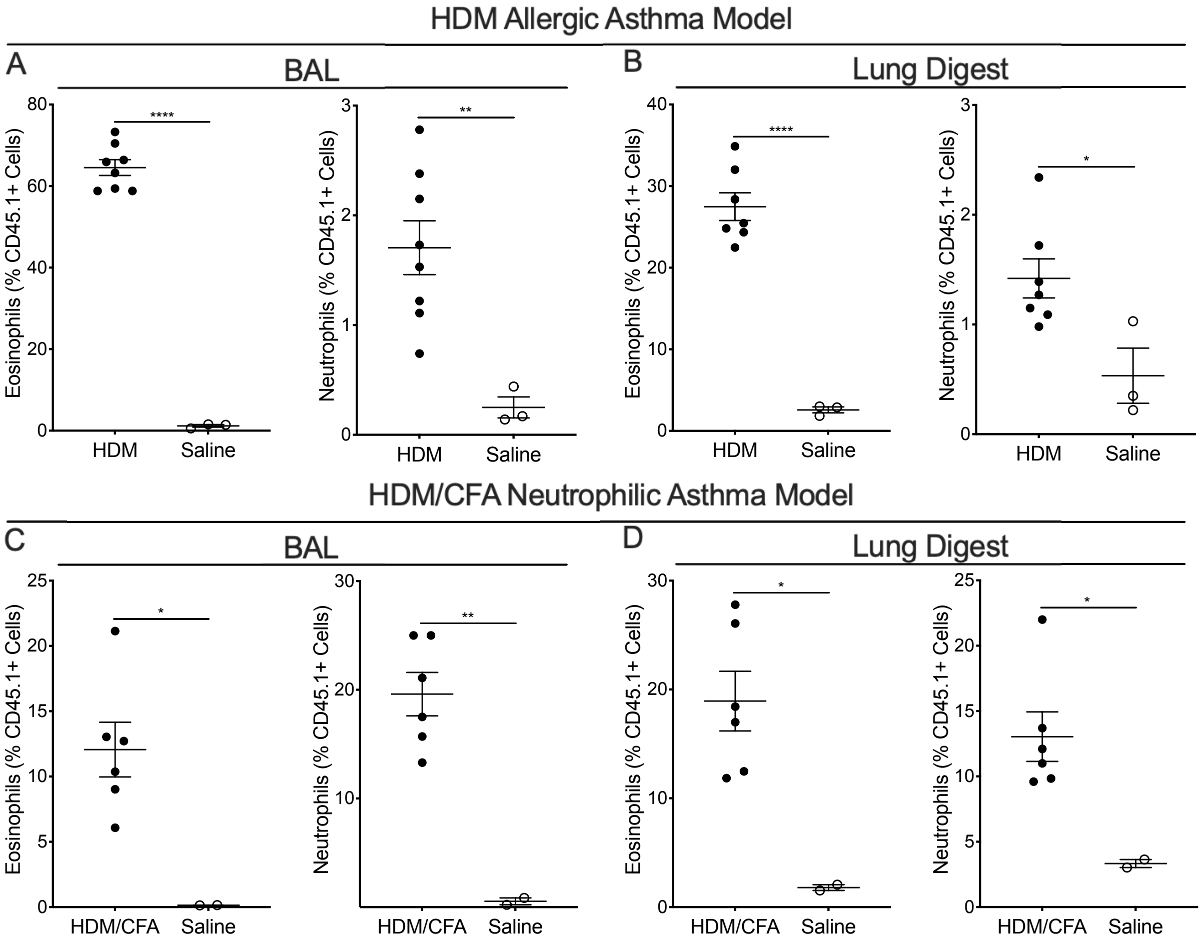

Supplemental Figure 2. We generated significant airway and lung eosinophilia in our house dust mite (HDM) allergic asthma model and mixed airway and lung eosinophilia and neutrophilia in our HDM/complete Freund's adjuvant (CFA) neutrophilic asthma model. (A, B) There was a significant increase in the percentage of both eosinophils and neutrophils in the bronchoalveolar lavage (BAL) fluid and digested lung tissue in our HDM allergic asthma model (expressed as a percentage of the total $CD45.1^{+}$ cells without exclusion of IV $CD45.2^{+}$ cells). (C, D) In contrast, we identified a larger increase in the percentage of airway and lung neutrophils and a less prominent increase in the percentage of airway and lung eosinophils in our HDM/CFA neutrophilic asthma model. Mean values are shown with error bars representing the standard error of the mean. For comparisons of individual cell population percentages from mice sensitized and challenged with either HDM or HDM/CFA versus saline controls, *P* values were calculated using an unpaired 2-tailed Student's *t* test. * indicates a *P* value <0.05, ** indicates a *P* value <0.01, *** indicates a *P* value <0.001, and **** indicates a *P* value <0.0001.

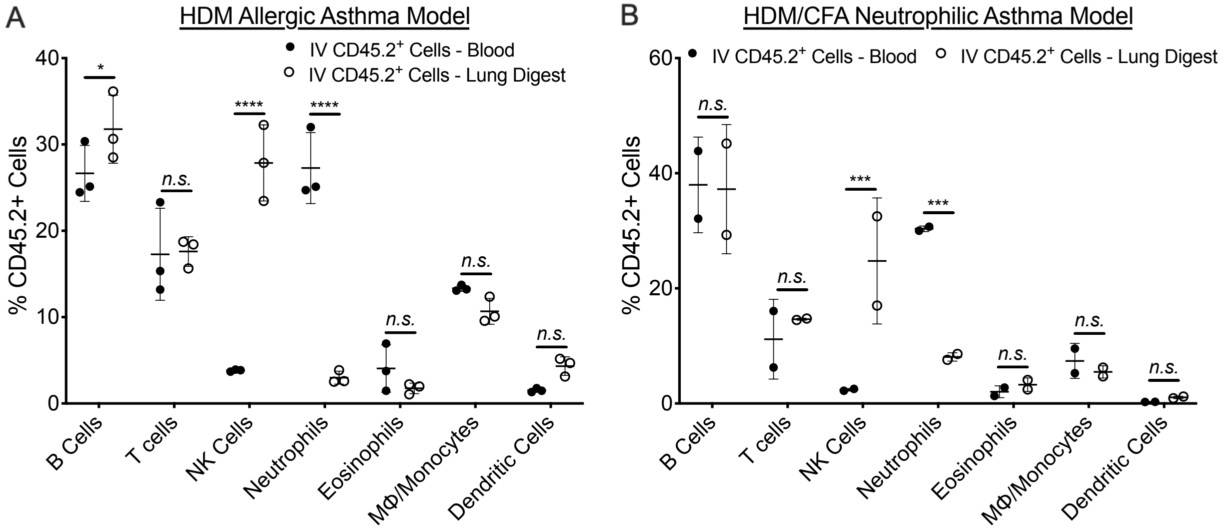

Supplemental Figure 3. The composition of intravascularly (IV) labeled leukocytes in the blood and digested lung tissue in two different models of asthma. (A) In mice receiving airway sensitization and challenge with saline in our HDM allergic asthma model, IV labeled leukocytes ($CD45.2^{+}$) identified in the blood had similar composition to IV labeled leukocytes in digested lung tissue, consisting primarily of lymphocyte populations and neutrophils, and we also saw a similar pattern in mice receiving sensitization and challenge with saline in our HDM/CFA neutrophilic asthma model (B). Mean values are shown with error bars representing the standard deviation. For comparisons of individual cell population percentages in the blood versus intravascular compartment of the lung tissue, *P* values were calculated using a 2-way ANOVA with correction for multiple comparisons using the two-stage step-up method of Benjamini, Krieger, and Yekutieli. * indicates a *P* value <0.05, ** indicates a *P* value <0.01, *** indicates a *P* value <0.001, and **** indicates a *P* value <0.0001.
